# Early diagnosis of brain metastases using cerebrospinal fluid cell‐free DNA‐based breakpoint motif and mutational features in lung cancer

**DOI:** 10.1002/ctm2.1221

**Published:** 2023-03-16

**Authors:** Xueting Qin, Yujun Bai, Shizhen Zhou, Hongjin Shi, Xiaoli Liu, Song Wang, Xiaoying Wu, Jiaohui Pang, Xi Song, Xiaojun Fan, Qiuxiang Ou, Yang Xu, Hua Bao, Li Li, Jun Li, Yang Shao, Shuanghu Yuan

**Affiliations:** ^1^ Department of Radiation Oncology Shandong Provincial Key Laboratory of Radiation Oncology Shandong Cancer Hospital and Institute Shandong First Medical University and Shandong Academy of Medical Sciences Jinan China; ^2^ Department of Radiation Oncology Tai'an Central Hospital (Tai'an Central Hospital Affiliated to Qingdao University Taishan Medical Care Center) Tai'an China; ^3^ Department of Neurosurgery Shandong Cancer Hospital and Institute Shandong First Medical University and Shandong Academy of Medical Sciences Jinan China; ^4^ Shandong Cancer Hospital Cheeloo College of Medicine Shandong University Jinan China; ^5^ Geneseeq Research Institute Nanjing Geneseeq Technology Inc Nanjing China; ^6^ Department of Biochemistry Zhongshan School of Medicine Sun Yat‐sen University Guangzhou China; ^7^ Department of Radiation Oncology The Affiliated Cancer Hospital of Zhengzhou University Zhengzhou China

Dear Editor,

In this study, we developed a sensitive machine learning model with a remarkable capacity to predict brain metastases (BM) in lung cancer patients using the breakpoint motif (BPM) features in cerebrospinal fluid (CSF) circulating tumour DNA (ctDNA). We have also assessed the mutational profile in CSF ctDNA, revealing promising BM‐related prognostic biomarkers in lung cancer patients.

BM is frequently associated with a short life expectancy and a high mortality rate in lung cancer patients.^1^ Early detection and timely treatment help to ameliorate the disease severity for lung cancer BM (LCBM). Brain magnetic resonance imaging (MRI) is the preferred method to evaluate the number, size and location of BM, but it lacks clear guidance to indicate the appropriate timing for screening. Cancer treatment may also obscure contrast enhancement, making the BM diagnosis more challenging.^2^ Meanwhile, CSF cytology provides valuable information about the pathologic conditions of cells involved in the central nervous system (CNS) and its coverings but is not sensitive enough for definitive diagnosis and highly relies on the pathologist's experience. Therefore, exploring sensitive and accurate methods is essential for promoting the early detection of LCBM.

Plasma cell‐free DNA (cfDNA) analysis has been widely adopted for assessing genomic features of cancer patients, monitoring response to treatment, quantifying minimal residual disease, and examining therapy resistance.^3^, ^4^, ^5^, ^6^, ^7^ Particularly, Guo et al. have leveraged the elastic‐net logistic regression algorithm to integrate the 6 bp BPM feature in plasma cfDNA and successfully built a sensitive model for stage I lung adenocarcinoma detection.^8^ As CSF ctDNA has been gaining credibility for its high capability of detecting somatic genetic alterations in patients with CNS malignancies,^9^ this study aims to develop a robust model for the sensitive detection of LCBM using genetic features derived from CSF ctDNA.

In this study, 76.6% of lung cancer patients (62/81) were diagnosed with parenchymal BM with or without other types of CNS diseases by enhanced brain MRI and/or computerized tomography (CT) scan (Table S1). CSF cytology was performed for 71 patients initially admitted to our hospital as a complementary approach for diagnosing leptomeningeal metastasis. All 81 patients underwent lumbar puncture to collect CSF for targeted next‐generation sequencing (NGS), followed by extraction of BPM and mutational features for modelling (Supplementary Material).

According to the BM status and the relationship with follow‐up time, the 81 patients were classified into three subgroups, including 62 POS patients (patients whose BM status was already positive at CSF sampling), 10 NEG patients (patients whose BM status was negative at CSF sampling and remained unchanged during the follow‐up) and nine NTP patients (patients whose BM status turned from negative at CSF sampling to positive during the follow‐up). As NTP patients were generally located between POS and NEG patients in the principal component analysis (Figure S1), we, therefore, assigned 70 patients with definitive BM status at CSF sampling (62 POS and eight randomly selected NEG) to the training cohort to develop the BM detection model and 11 patients (nine NTP and two randomly selected NEG) to the testing cohort for independent evaluation of the model performance (Figure 1A).

**FIGURE 1:**
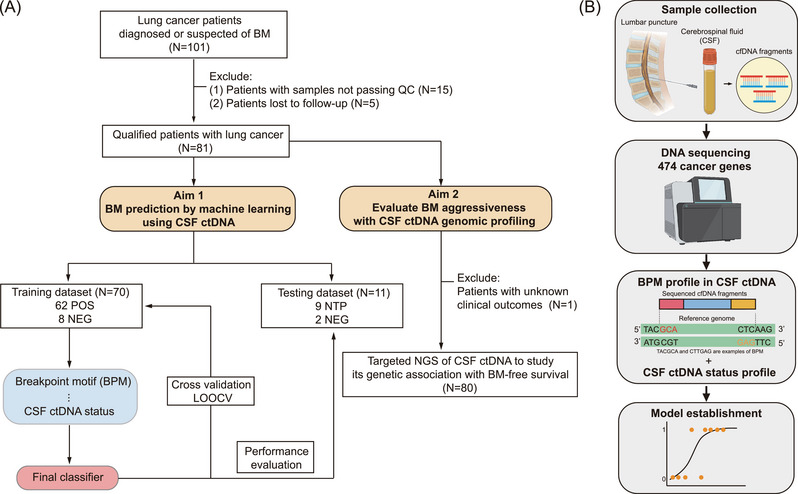
Schematic demonstration of the study design. (A) After excluding 15 patients with CSF samples that failed to pass the quality control (QC) examination and five patients lost to follow‐up, sequencing data of 81 patients were analyzed to assess the potential of cerebrospinal fluid (CSF) circulating tumour DNA (ctDNA) in brain metastases (BM) prediction. Based on the BM status, these 81 patients were categorized into three subgroups: NEG, NTP and POS. The BM predictive model was developed based solely on the breakpoint motif (BPM) profile (BPM model) or combined CSF ctDNA status and BPM profile (integrated model). To train the machine learning model, 62 POS and eight randomly selected NEG patients were recruited into the training dataset. Leave‐one‐out cross‐validation (LOOCV) was performed to evaluate the predictive performance of the BM predictive model, and the area under the curve (AUC) was calculated based on the holdout predictions. Independent testing of the model's performance was performed in the testing cohort comprising nine NTP and two randomly selected NEG patients. A higher predicted score indicates a higher probability of developing BM. Meanwhile, targeted next‐generation sequencing (NGS) data of CSF ctDNA in 80 patients with known clinical outcome data were assessed to reveal genetic alterations related to BM aggressiveness. (B) Schematic demonstration of the integrated model construction. CSF samples were obtained from each participant through the lumbar puncture. A total of 474 cancer‐related genes in CSF ctDNA were sequenced using high‐throughput targeted NGS. The BPM profile was combined with each patient's CSF ctDNA status to generate the integrated machine‐learning model using the elastic‐net logistic regression algorithm.

Since the predictive model built solely on CSF ctDNA status showed a relatively high false‐positive rate in detecting LCBM (Figure S2), we wondered if incorporating the ctDNA status feature into the model based on BPM features of CSF ctDNA using elastic‐net logistic regression, hereafter referred to as “integrated model”, could help improve the model performance (Figure 1B). In the training cohort, the integrated model achieved an area under the curve (AUC) of 0.940 (95% confidence interval [CI]: 0.885–0.995), which was slightly better than the BPM model with an AUC of 0.929 (95% CI: 0.862–0.997, Figure 2A). Both models performed similarly in distinguishing lung cancer patients with different BM or ctDNA status (Figure 2B,C and Figure S3A,B). Furthermore, both models were tested against different patient subgroups and persisted in high performance regardless of patients’ clinical characteristics, such as age, ctDNA status, smoking and treatment history (Figure 2D and Figure S3C). At 90% sensitivity, comparable high specificities were achieved by both models when tested in the matched cohorts (Figure 2E and Figure S3D).

**FIGURE 2:**
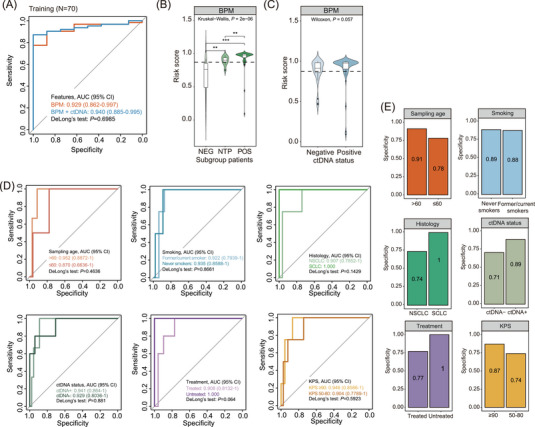
Development of the BPM model. (A) ROC curves evaluating the performance of brain metastases (BM) predictive models (BPM and integrated) in distinguishing BM‐positive patients from BM‐negative patients in the training cohort (N = 70). (B and C) Violin plots illustrating the BM risk score distribution in patient subgroups classified based on BM status (B) or ctDNA status (C) built on the BPM model. The optimal cutoff for the risk score was 0.8714, as shown by the dotted line. (D) ROC curves evaluating the performance of the BPM model in specified subgroup patients of the training dataset (N = 70). *P* values indicate the significance levels of the model's performance in two matched cohorts computed by DeLong's test. (E) Bar plots illustrating the diagnostic specificities of the BPM model at 90% sensitivity for subgroup patients, comparing patients’ sampling age, smoking history, histology, ctDNA status, treatment history and KPS.

Next, we assessed our models’ performance in the testing cohort comprising 9 NTP and 2 NEG patients. Interestingly, the integrated model achieved an AUC of 0.833 (95% CI: 0.4681–1), whereas the BPM model performed slightly better, with an AUC of 0.944 (95% CI: 0.7905–1, Figure 3A). The lower AUC of the integrated model might be explained by the inclusion of three BM‐negative patients who tested positive for CSF ctDNA (two for training and one for testing). The positive CSF ctDNA result might be because CSF ctDNA results indicate BM status earlier than conventional neurological imaging, either because genomic changes have not yet caused organic pathologic changes or because the organic disease cannot be detected at an early stage.

**FIGURE 3:**
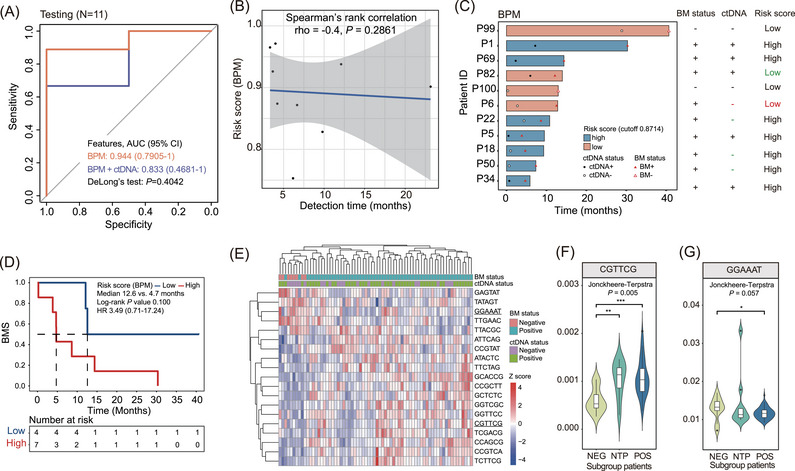
Evaluation of the breakpoint motif (BPM) model. (A) Receiver operating characteristics (ROC) curves evaluating the performance of brain metastases (BM) predictive models in distinguishing BM‐positive patients from BM‐negative patients in the testing cohort (*N* = 11). (B) Correlation plot evaluating the relationship between the risk score computed by the BPM model and BM detection time in the testing cohort. (C) Schematic demonstration of the lead time of the BPM model in distinguishing high‐risk patients from low‐risk patients for each subject in the testing cohort. Patients were classified into two subgroups based on the cutoff value of 0.8714 of the BPM model obtained from the training cohort. Green colour labels wrong predictions by cerebrospinal fluid (CSF) circulating tumour DNA (ctDNA) status or the BPM model. Red colour labels wrong predictions by both screening methods. (D) Kaplan Meier curves estimating the BM‐free survival (BMS) of patients in the testing cohort with a high risk of developing BM compared to those with low risk computed by the BPM model. (E) Hierarchical clustering analysis of BPM with non‐zero coefficients in the training cohort. Underlined motifs had the greatest positive and negative coefficients in the model. (F and G) Violin plots showing frequencies of the CGTTCG motif (F) and the GGAAAT motif (G) in three patient subgroups categorized based on patients’ BM status (*N* = 81). One‐sided Jonckheere‐Terpstra tests were used to test the trend in ordered patient cohorts (in order of NEG, NTP and POS).

While not statistically significant, higher risk scores were associated with shorter BM detection times in both models (Figure 3B and Figure S4A). It is worth noting that the BPM model not only distinguished all seven high‐risk patients from low‐risk but also outperformed the integrated model for its lower false‐negative rates in predicting BM of low‐risk patients (BPM model: 50% versus integrated model: 66.7%; Figure 3C and Figure S4B). Additionally, the risk score computed by the BPM model better reflected BM‐free survival (BMS) than the integrated model (Figure 3D and Figure S4C). Overall, these findings suggested that the BPM model performs better in predicting LCBM. Incorporating the CSF ctDNA status feature into the BPM model could not further improve the model's performance.

To determine which motif contributed mostly to the model's predictive power, we performed a hierarchical clustering analysis in the training cohort using motifs with non‐zero coefficients in the BPM model (Figure 3E). The CGTTCG motif was found to have the most positive coefficient, which showed an upward trend in three patient subgroups categorized by BM status (Figure 3F). In contrast, the GGAAAT motif, which had the greatest negative coefficient, presented an opposite trend in these patients (Figure 3G). In the testing cohort, a similar distribution pattern of the CGTTCG motif was observed (Figure S5). However, GGAAAT did not show the expected trend due to the limited sample size.

Lastly, we performed comprehensive genomic profiling using CSF ctDNA mutational features to identify BM‐associated genetic alterations in 80 lung cancer patients with known clinical outcomes. The most frequent genomic alterations were in the *EGFR*, *TP53*, *RB1*, *CDKN2A*, and *CDKN2B* genes (Figure 4A). Noteworthy, we emphasized alterations in the DNA‐damage response (DDR)‐related pathways for their role in leptomeningeal metastasis development.^10^ At univariate analysis, *RB1* variants, *EGFR* amplification, and the Fanconi Anemia (FA) pathway alterations were individually associated with BMS (Figure 4B–G and Table S2). *RB1* variants and *EGFR* amplification in CSF ctDNA of lung cancer patients remained independently associated with an inferior prognosis in the multivariate model (*P* = 0.028 and 0.023, respectively; Figure 4H).

**FIGURE 4:**
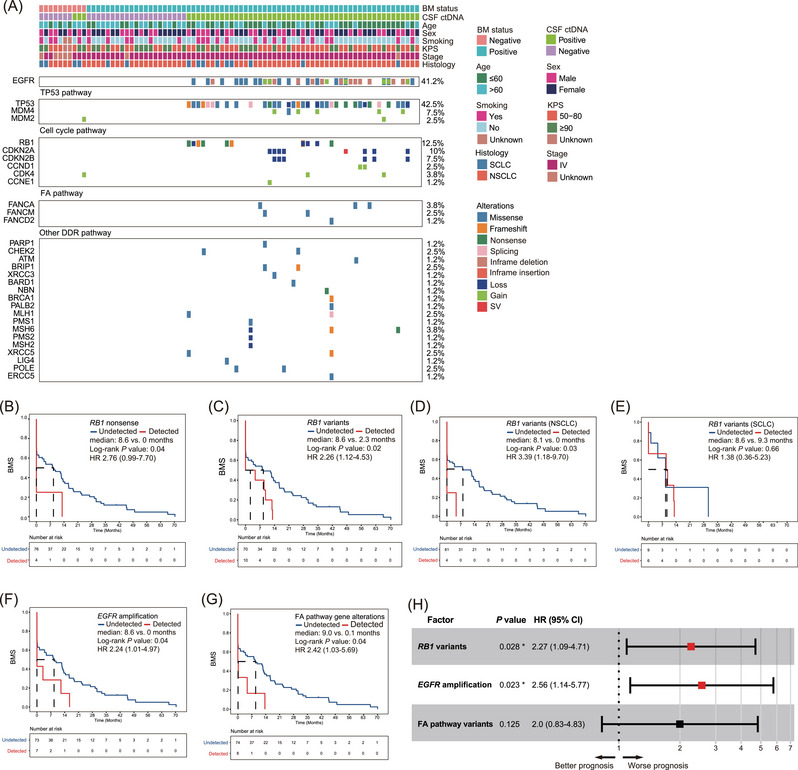
Identifying brain metastases (BM)‐associated genetic alterations using cerebrospinal fluid (CSF) circulating tumour DNA (ctDNA). (A) Co‐mutation plot of lung cancer patients with confirmed or suspected BM (*N* = 80). Patients’ clinical characteristics are shown on the top, while the top frequently mutated gene alterations are grouped by oncogenic pathways previously reported to be associated with LCBM. (B) Kaplan Meier estimates of BM‐free survival (BMS) in lung cancer patients harbouring *RB1* nonsense mutations compared to those without detectable *RB1* nonsense mutations. (C) BMS in lung cancer patients harbouring *RB1* variants (including SNVs and CNVs) compared to patients without detectable *RB1* variants. (D and E) BMS in two stratified patient cohorts comprising either NSCLC (D) or SCLC patients (E) with co‐occurring *RB1* variants compared to corresponding control patients. (F) BMS in lung cancer patients harbouring *EGFR* copy‐number gain variants compared to patients without detectable *EGFR* amplification. (G) BMS in lung cancer patients harbouring Fanconi anaemia (FA) pathway genetic alterations compared to others. (H) Multivariate analysis of genetic factors associated with BM. FA pathway variants include missense mutations in *FANCA*, *FANCM* and *FANCD2*. Asterisks represent significant *P‐*values based on the Cox regression model.

As a proof‐of‐concept pilot study exploring the clinical application of BPM profiling in the sensitive detection of BM with a machine‐learning model in lung cancer patients, our study has a few limitations. The limited sample size may potentially compromise the credibility of our BM predictive model. Expanding the cohort size is warranted to improve the statistical power of a more accurate estimation of the risk score in lung cancer patients. In addition, although most patients in our study developed parenchymal BM during progression, the study cohort comprises various BM types due to sample availability. The cfDNA and BPM profiles may differ and need to be further investigated. Therefore, we plan to conduct a more extensive study and develop a BPM model capable of identifying patients with different BM types, which may add significant value to the current model for its clinical utility.

In summary, we established a robust BM predictive model using the BPM features of CSF ctDNA and profiled genomic alterations associated with BM in lung cancer patients. Our study provides insights into the potential use of CSF ctDNA sequencing for the early detection of LCBM and disease management.

## CONFLICT OF INTEREST STATEMENT

Song Wang, Xiaoying Wu, Jiaohui Pang, Xi Song, Xiaojun Fan, Qiuxiang Ou, Yang Xu, Hua Bao and Yang Shao are employees of Nanjing Geneseeq Technology Inc. The remaining authors declare no conflict of interest.

## Supporting information

Supporting Information
