# Supplementary material for: Early diagnosis of brain metastases using cerebrospinal fluid cell‐free DNA‐based breakpoint motif and mutational features in lung cancer
Source: Clin Transl Med. 2023 Mar 16;13(3):e1221. doi: 10.1002/ctm2.1221 (PMC10019768; doi:10.1002/ctm2.1221)
Supplement: Supplementary file 1 — Supporting Information [file CTM2-13-e1221-s001.docx]

**Supplementary Information**

**Early diagnosis of brain metastases using cerebrospinal fluid cell-free DNA-based breakpoint motif and mutational features in lung cancer**

Manuscript type: Letter-to-Editor

Xueting Qin^1^, Yujun Bai^2^, Shizhen Zhou^3^, Hongjin Shi^1^, Xiaoli Liu^4^, Song Wang^5^, Xiaoying Wu^5^, Jiaohui Pang^5^, Xi Song^5^, Xiaojun Fan^5^, Qiuxiang Ou^5^, Yang Xu^5^, Hua Bao^5^, Li Li^1^, Jun Li^6^, Yang Shao^5^, Shuanghu Yuan*^1,4,7^

^1^Department of Radiation Oncology, Shandong Provincial Key Laboratory of Radiation Oncology, Shandong Cancer Hospital and Institute, Shandong First Medical University and Shandong Academy of Medical Sciences, Jinan, Shandong, China

^2^Department of Radiation Oncology, Tai'an Central Hospital (Tai'an Central Hospital Affiliated to Qingdao University, Taishan Medical Care Center), Tai'an, Shandong, 271000, China

^3^Department of Neurosurgery, Shandong Cancer Hospital and Institute, Shandong First Medical University and Shandong Academy of Medical Sciences, Jinan, Shandong, China

^4^Shandong Cancer Hospital, Cheeloo College of Medicine, Shandong University, Jinan, Shandong, China

^5^Geneseeq Research Institute, Nanjing Geneseeq Technology Inc. Nanjing, Jiangsu, China

^6^Department of Biochemistry, Zhongshan school of medicine, Sun Yat-sen University, Guangzhou, Guangdong, China

^7^Department of Radiation Oncology, The Affiliated Cancer Hospital of Zhengzhou University, Zhengzhou, Henan, China

***Corresponding author:**

Shuanghu Yuan, MD, PhD

Phone: 86-13853106916

Fax: 86-531-87984079

Email: [yuanshuanghu@sina.com](mailto:yuanshuanghu@sina.com)

ORCID: https://orcid.org/0000-0002-8327-2524

## Materials and Methods

### Patients and CSF collection

Criteria for selecting subjects eligible to include in this study were as follows: 1) patients who were initially diagnosed with lung cancer by histological examination between June 2019 and October 2021; 2) patients with confirmed or suspected BM; 3) patients without contraindication for lumbar puncture. BM status was confirmed by two experienced neuropathologists using brain MRI and/or brain CT. The study was approved by the Ethics Committee of the Shandong Cancer Hospital and Institute (SDTHEC2020004042). Written consent form was collected from each patient before sample collection. Within 2 hours of CSF collection, 14 mL of CSF was centrifuged at 1,000x g at 4°C for 5 min. The supernatant was aseptically transferred to prelabeled cryotubes and subjected to targeted next-generation sequencing (NGS).

### Cell-free DNA extraction

CSF samples were sent to a Clinical Laboratory Improvement Amendments (CLIA)-certified and College of American Pathologists (CAP)-accredited clinical testing laboratory (Nanjing Geneseeq Technology Inc.) for the following analyses. cfDNA extraction was performed based on the manufacturer’s protocol for the QIAamp Circulating Nucleic Acid Kit (Qiagen Cat. No. 55114). Briefly, CSF samples were first centrifuged using two-step centrifugation at 4°C, including 1,900x g for 10 min and then 16,000x g for 10 min. This step removes residual precipitated cellular components and various particles. 5 mL of CSF was mixed with 500 μL of proteinase K and 4 mL of buffer ACL. After incubation in a 60°C water bath for 30 min, 9 mL of buffer ACB was added, and the mixture was incubated on ice for 5 min. The mixture was then filtered through the QIAamp Mini columns using the Vacuum Pump (Laboport, N840.3). The columns with DNA attached to the membrane were washed with ACW1, ACW2, and ethanol. DNA was eluted in 50 μL of nuclease-free water. As a normal control, the genomic DNA of white blood cells in sediments was extracted using the DNeasy Blood and Tissue Kit (Qiagen Cat. No. 69504). The extracted DNA samples were quantified on Qubit 3.0 fluorometer using the dsDNA HS Assay Kit (Invitrogen Q32850) and qualified using Agilent Bioanalyzer 2100.

### Targeted capture and sequencing

Sequencing libraries were prepared for Illumina sequencing using the KAPA Hyper Prep Kit (KAPA Biosystems) according to the manufacturer’s instructions. Compared to third-generation sequencing, such as Oxford Nanopore Technology, Illumina sequencing with well-established platforms and pipelines is better suited for short read-based liquid biopsy sequencing. Targeted capture enrichment was performed as previously described [[1](#_ENREF_1)]. In brief, the Radiotron^®^ panel (Nanjing Geneseeq Technology Inc.) covering 474 critical cancer-related genes with a total genomic region of 130 kb was used to identify genomic variants in hotspot exons and regions [[2](#_ENREF_2)]. According to its internal specifications, the resulting sequencing panel can identify somatic mutations with a sensitivity of 98% and a positive predictive value (PPV) of 95% [[3-5](#_ENREF_3)]. Library fragment size was determined on Bioanalyzer 2100 (Agilent Technologies). The targeted enriched library was then sequenced on Illumina HiSeq 4000 platform (Illumina, San Diego, CA, USA) according to the manufacturer’s instructions.

### Mutation calling

Raw sequencing data were analyzed by a validated automation pipeline. Specifically, raw data were first demultiplexed and subjected to FASTQ file quality control using Trimmomatic [[6](#_ENREF_6)]. Only data without extra nucleotide bases and passed quality control (QC above 15) were retained. According to the current CAP guidelines, qualified raw reads were mapped to chromosomal positions and reference/alternate alleles based on the HG19 human reference genome using Burrows-Wheeler Aligner (BWA-mem, v0.7.12; <https://github.com/lh3/bwa/tree/master/bwakit>). Local realignment around the insertions/deletions (INDELs) and base quality score recalibration were applied using the Genome Analysis Toolkit (GATK 3.4.0; <https://software.broadinstitute.org/gatk/>). PCR duplicates were removed using Picard. Genetic alterations were called by VarScan2 [[7](#_ENREF_7)] with the following parameters: 1) for somatic mutations: altered reads ≥3 (depth ≥50X), 2) for structural variants (SVs): split-read ≥3, 3) for copy-number gain variants: *MET* and *ERBB2* gene ratio ≥1.8, other genes ≥2, 4) for copy-number loss variants: *CDKN2A* and *CDKN2B* gene ratio ≤0.25, other genes ≤0.6.

### Development and evaluation of the BM predictive model

We extracted the CSF ctDNA status and BPM feature profile from targeted NGS data for BM predictive modeling. The extraction of the BPM profile has been previously described [[8](#_ENREF_8)]. Briefly, the BPM profiled the 3bp genomic DNA sequences upstream and downstream of 5’ end breakpoints after aligning the sequencing reads to the human reference genome HG19. For each sample, a total of 4096 (4^6^) 6-mer motifs were extracted, and the frequency of each motif was calculated. As a result, for N samples, an N×4096 matrix was generated and used alone (4,096 features) or combined with ctDNA detection status (4,097 features) as the input to construct elastic*-*net logistic regression models to predict BM.

The 81 patients were classified into three subgroups according to their BM status and its relationship with sampling time, including 62 POS patients (patients whose BM status was already positive at CSF sampling), 10 NEG patients (patients whose BM status was negative at CSF sampling and remained unchanged during the follow-up) and 9 NTP patients (patients whose BM status turned from negative at CSF sampling to positive during the follow-up). The training dataset recruited 70 patients whose BM status at CSF sampling was definitive, including 62 POS and 8 randomly selected NEG patients. The testing dataset consisted of 11 patients, including 9 NTP and 2 randomly selected NEG patients.

We used two approaches to evaluate the predictive performance of the BPM profile, leave-one-out cross-validation (LOOCV) and independent testing. Firstly, LOOCV was used in the training cohort to assess the predictive performance; thus, for 70 patients, 70 holdout predictions (probability of BM) could be obtained, and AUC was calculated based on the holdout predictions. Then, the model derived from the training cohort was applied to the testing cohort for independent testing. The BM predictive model was developed based solely on the BPM profile (BPM model) or combined CSF ctDNA status and BPM profile (integrated model). For testing, a higher predicted score indicates a higher probability of developing BM. Patients were stratified into high-risk and low-risk groups based on the value maximizing the Youden index of the training model. Model construction and evaluation were performed in R (version 4.1.3) using the “caret” and “glmnet” packages.

### Statistical analysis

All statistical analyses were performed using R (version 4.1.3). Fisher's exact test was used to compare the clinical data of subgroup patients. Wilcoxon rank sum and Kruskal-Wallis tests were used to compare risk scores in patient subgroups classified based on CSF ctDNA status and BM status, respectively. Cochran Armitage tests and Jonckheere-Terpstra tests were used to test if a trend was observed in ctDNA detection rate and the frequencies of breakpoint motifs in ordered patient cohorts (in order of NEG, NTP, and POS), respectively. The ROC curves were generated using the “pROC” package in R. Based on true positive (TP), true negative (TN), false positive (FP) and false negative (FN) values, the sensitivity [TP/(TP+FN)], specificity [TN/(TN+FP)], positive predictive value (PPV), and negative predictive value (NPV), as well as the corresponding 95% confidence intervals (95% CI) of the model can be computed using the “epiR” package in R.

Brain metastasis-free survival (BMS) was defined as the time from the initial diagnosis of lung cancer to the incidence of BM diagnosed by neurological imaging approaches. Patients who had not developed BM were censored on the date of the last follow-up. Kaplan-Meier curves were used to compare the BMS of various patient groups, and the statistical difference was analyzed using the log-rank test. The hazard ratio (HR) and 95% CI were computed by the Cox regression model. The BM detection time was defined as the time from the CSF sampling to BM diagnosis. The correlation between the risk score and BM detection time was computed using Spearman’s rank correlation coefficient. A two-sided *P* value of less than 0.05 was considered statistically significant for all tests unless indicated otherwise (**P*<0.05, ***P*<0.01, and ****P*<0.001).

## References

1. Fang, W., Y. Ma, J.C. Yin, S. Hong, H. Zhou, A. Wang, et al., *Comprehensive Genomic Profiling Identifies Novel Genetic Predictors of Response to Anti-PD-(L)1 Therapies in Non-Small Cell Lung Cancer.* Clin Cancer Res, 2019. **25**(16): p. 5015-5026.

2. Wang, X., N. Yu, G. Cheng, T. Zhang, J. Wang, L. Deng, et al., *Prognostic value of circulating tumour DNA during post-radiotherapy surveillance in locally advanced esophageal squamous cell carcinoma.* Clin Transl Med, 2022. **12**(11): p. e1116.

3. Clifton K, R.T., Parseghian C, Raymond VM, Dasari A, Pereira AAL, Willis J, Loree JM, Bauer TM, Chae YK, Sherrill G, Fanta P, Grothey A, Hendifar A, Henry D, Mahadevan D, Nezami MA, Tan B, Wainberg ZA, Lanman R, Kopetz S, Morris V., *Identification of Actionable Fusions as an Anti-EGFR Resistance Mechanism Using a Circulating Tumor DNA Assay.* JCO Precis Oncol., 2019.

4. Jin, Y., H. Bao, X. Le, X. Fan, M. Tang, X. Shi, et al., *Distinct co-acquired alterations and genomic evolution during TKI treatment in non-small-cell lung cancer patients with or without acquired T790M mutation.* Oncogene, 2020. **39**(9): p. 1846-1859.

5. Shu, Y., X. Wu, X. Tong, X. Wang, Z. Chang, Y. Mao, et al., *Circulating Tumor DNA Mutation Profiling by Targeted Next Generation Sequencing Provides Guidance for Personalized Treatments in Multiple Cancer Types.* Sci Rep, 2017. **7**(1): p. 583.

6. Bolger, A.M., M. Lohse, and B. Usadel, *Trimmomatic: a flexible trimmer for Illumina sequence data.* Bioinformatics, 2014. **30**(15): p. 2114-20.

7. Reble, E., C.A. Castellani, M.G. Melka, R. O'Reilly, and S.M. Singh, *VarScan2 analysis of de novo variants in monozygotic twins discordant for schizophrenia.* Psychiatr Genet, 2017. **27**(2): p. 62-70.

8. Ma, X., Y. Chen, W. Tang, H. Bao, S. Mo, R. Liu, et al., *Multi-dimensional fragmentomic assay for ultrasensitive early detection of colorectal advanced adenoma and adenocarcinoma.* J Hematol Oncol, 2021. **14**(1): p. 175.

## Supplementary Figures


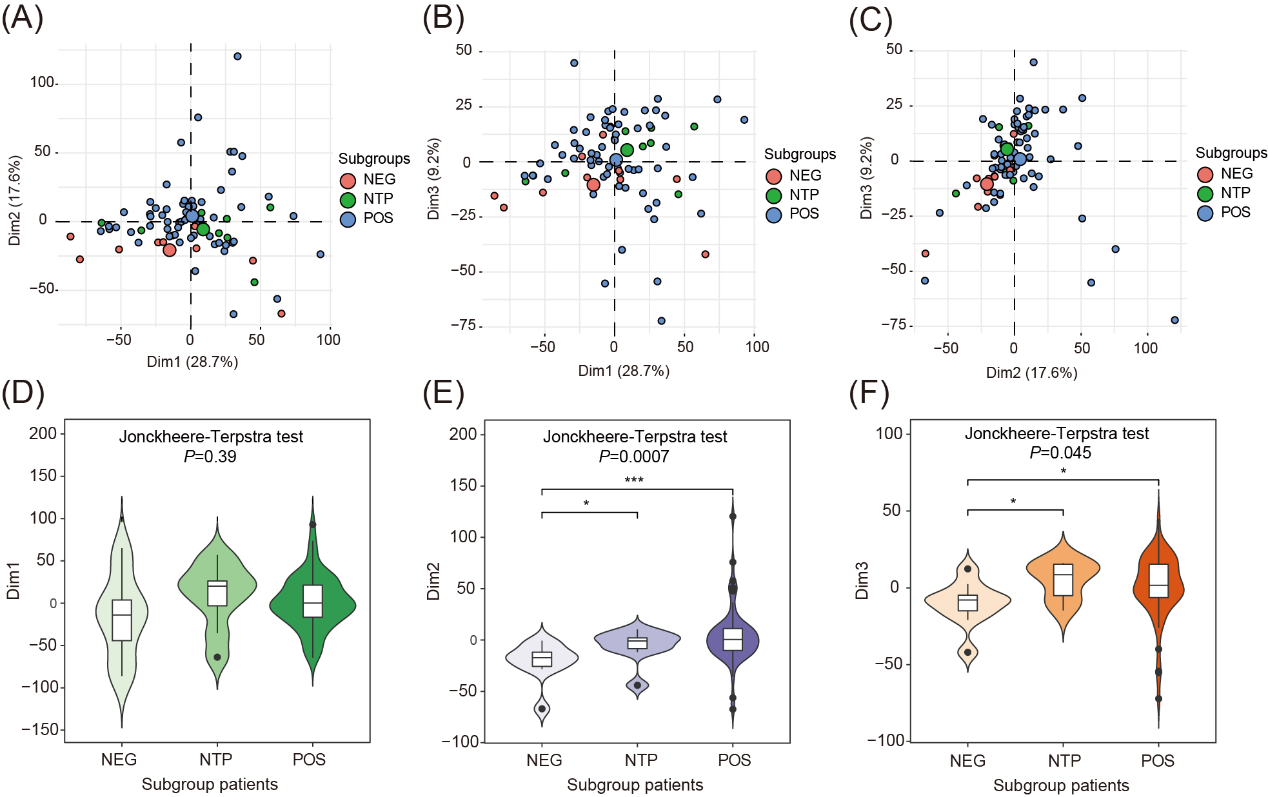


### Figure S1. PCA analysis.

(A-C) Principle component analysis (PCA) plots evaluating the separation of three patient subgroups (NEG, NTP, POS) in dimensions (Dim) of interest. (D-F) Violin plots illustrating Dim scores of three patient subgroups. One-sided Jonckheere-Terpstra tests were used for testing the trend in ordered patient cohorts (in order of NEG, NTP, and POS). **P*<0.05, ***P*<0.01, and ****P*<0.001 (Kruskal-Wallis test).


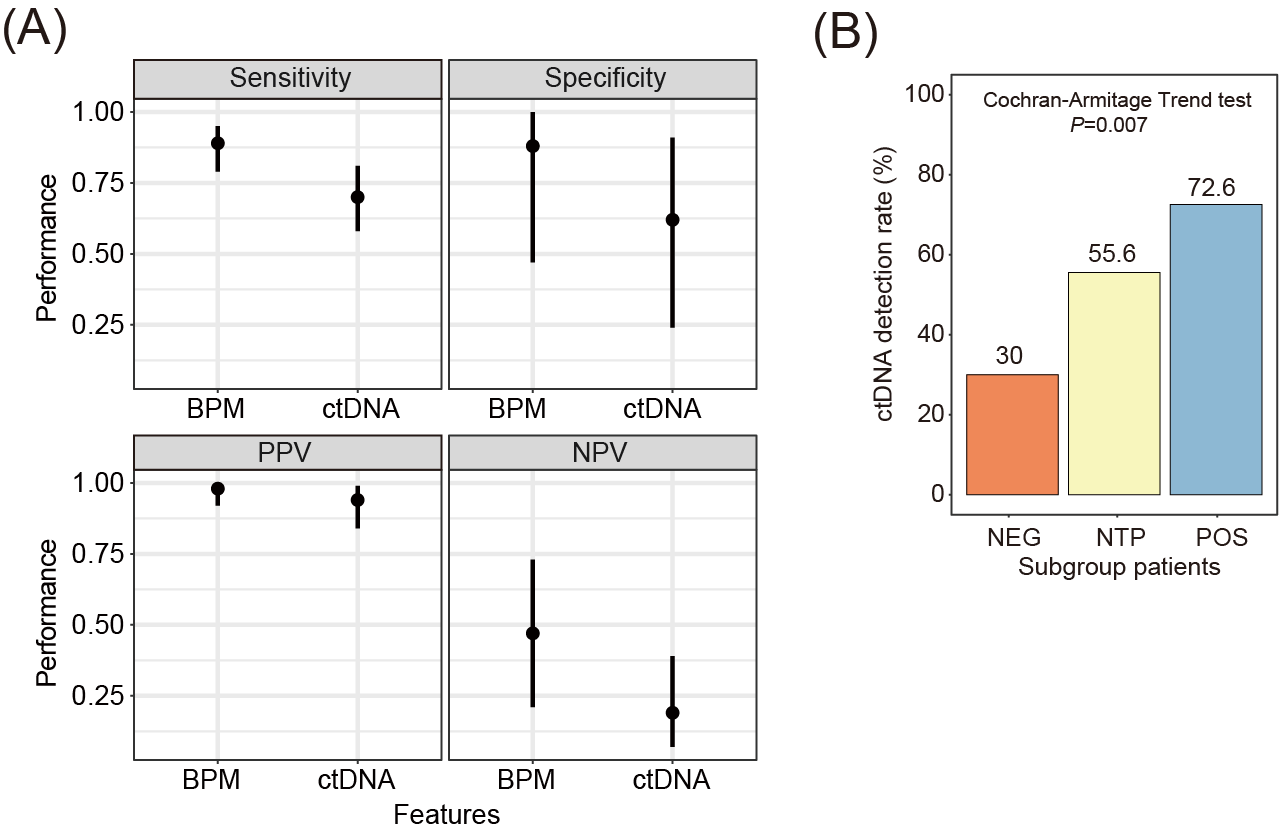


### Figure S2. Model built solely on CSF ctDNA status or BPM features.

(A) Performance of BM predictive models built solely on patients' BPM or CSF ctDNA status features in distinguishing BM-positive patients from BM-negative patients (N=81). The error bars represent the 95% confidence interval. (B) Bar plots showing the CSF ctDNA detection rates in three patient subgroups.


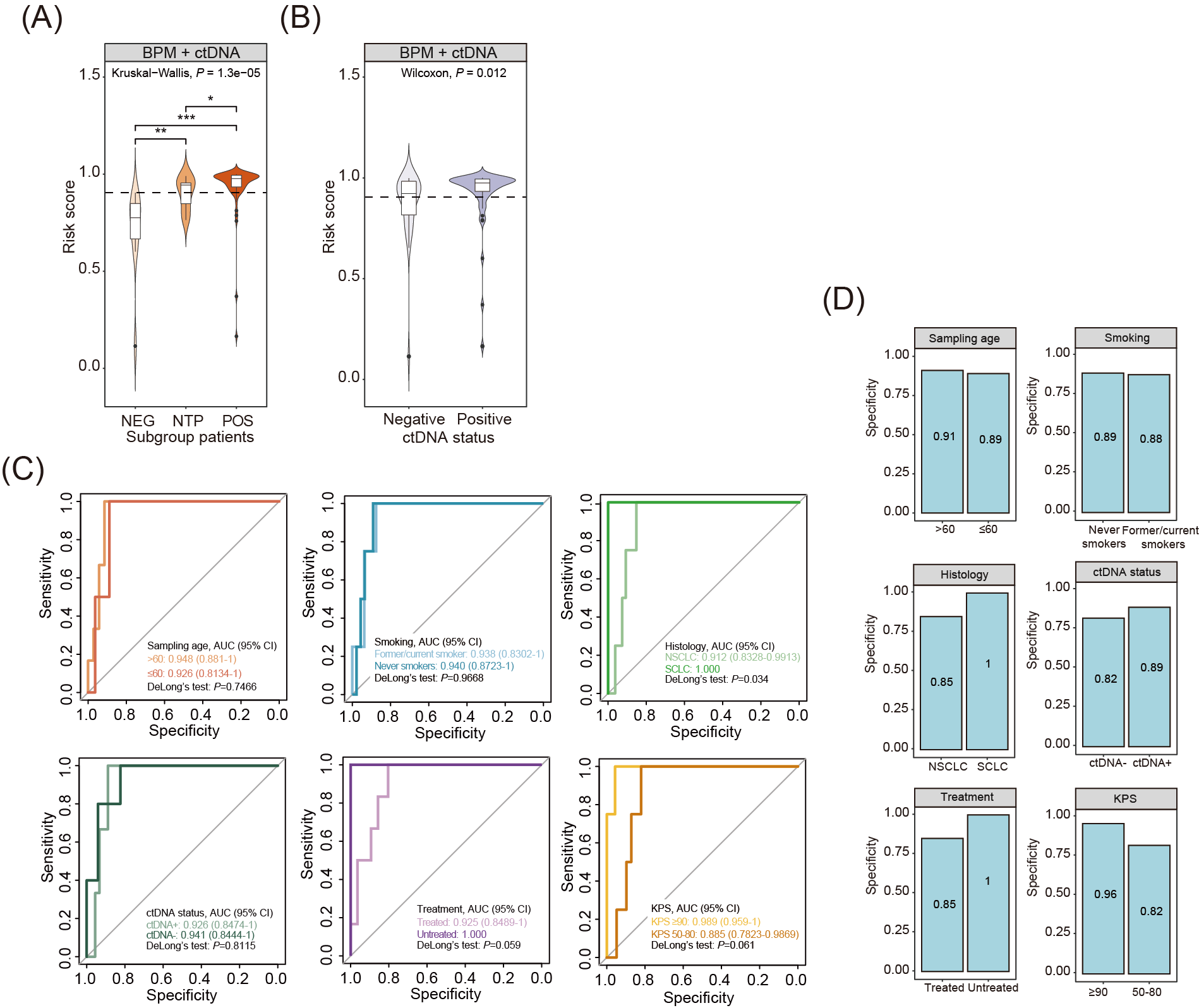


### Figure S3. Development of the integrated model.

(A and B) Violin plots illustrating the BM risk score distribution in patient subgroups classified based on BM status (A) or ctDNA status (B) built on the integrated model. The optimal cutoff for the risk score was 0.9058, as shown by the dotted line. **P*<0.05, ***P*<0.01, and ****P*<0.001 (Kruskal-Wallis test). (C) ROC curves evaluating the performance of the integrated model in specified subgroup patients of the training dataset (N=70). *P* values indicate the significance levels of the model’s performance in two matched cohorts computed by DeLong’s test. (D) Bar plots illustrating the diagnostic specificities of the integrated model at 90% sensitivity for subgroup patients, comparing patients’ sampling age, smoking history, histology, ctDNA status, treatment history, and KPS.


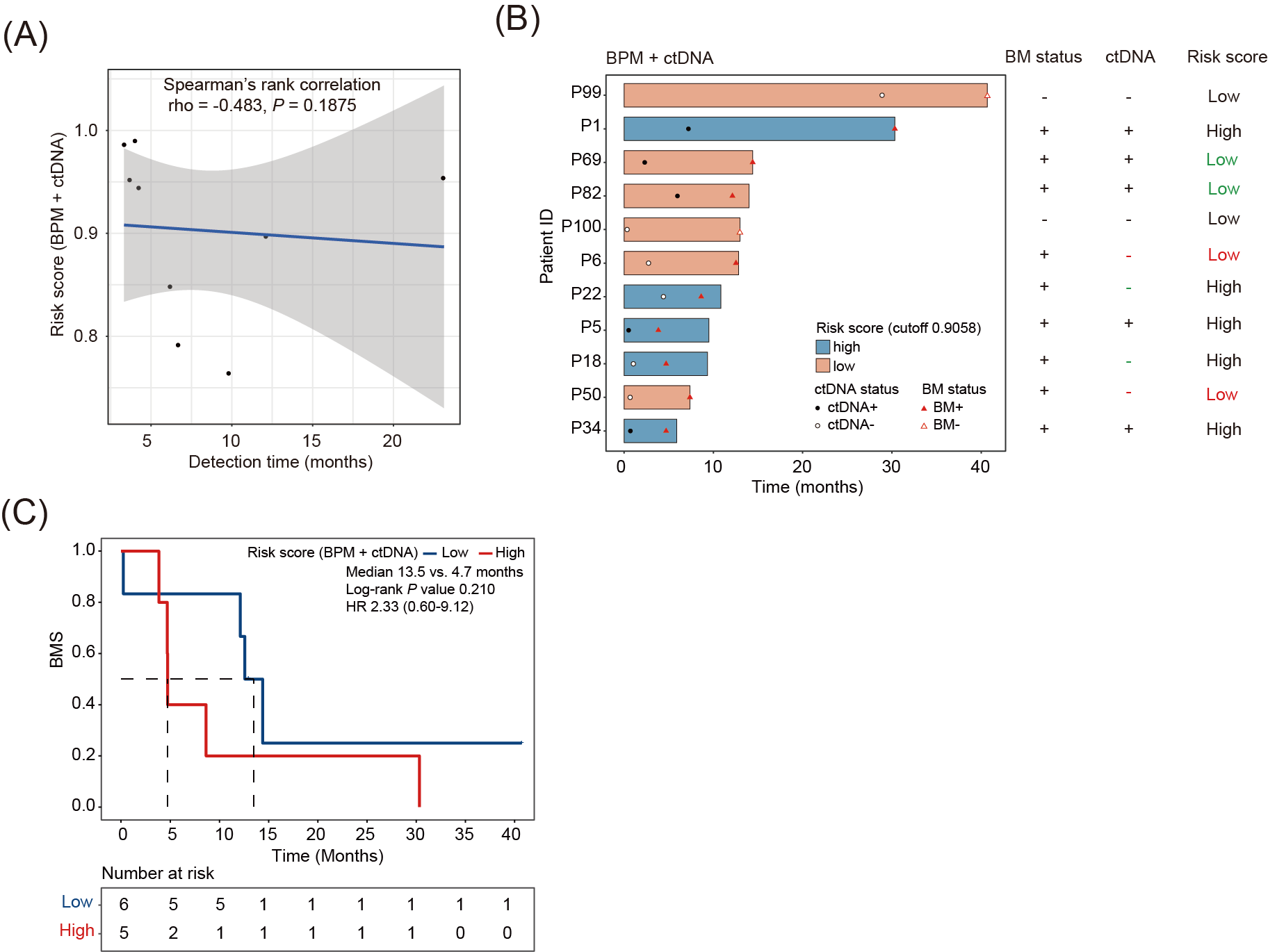


### Figure S4. Evaluation of the integrated model performance.

(A) Correlation plot demonstrating the association between the risk score computed by the integrated model and the BM detection time in the testing cohort (N=11). (B) Schematic demonstration of the detection time of the integrated model in BM prediction for each patient in the testing cohort. Patients were classified into high-risk or low-risk subgroups based on the cutoff value of 0.9058 obtained from the training cohort. Green labels wrong predictions by either CSF ctDNA status or the integrated model. Red labels wrong predictions by both screening methods. (C) Kaplan Meier curves estimating the BMS of patients in the testing cohort with a high risk of developing BM compared to those with low-risk computed by the integrated model.


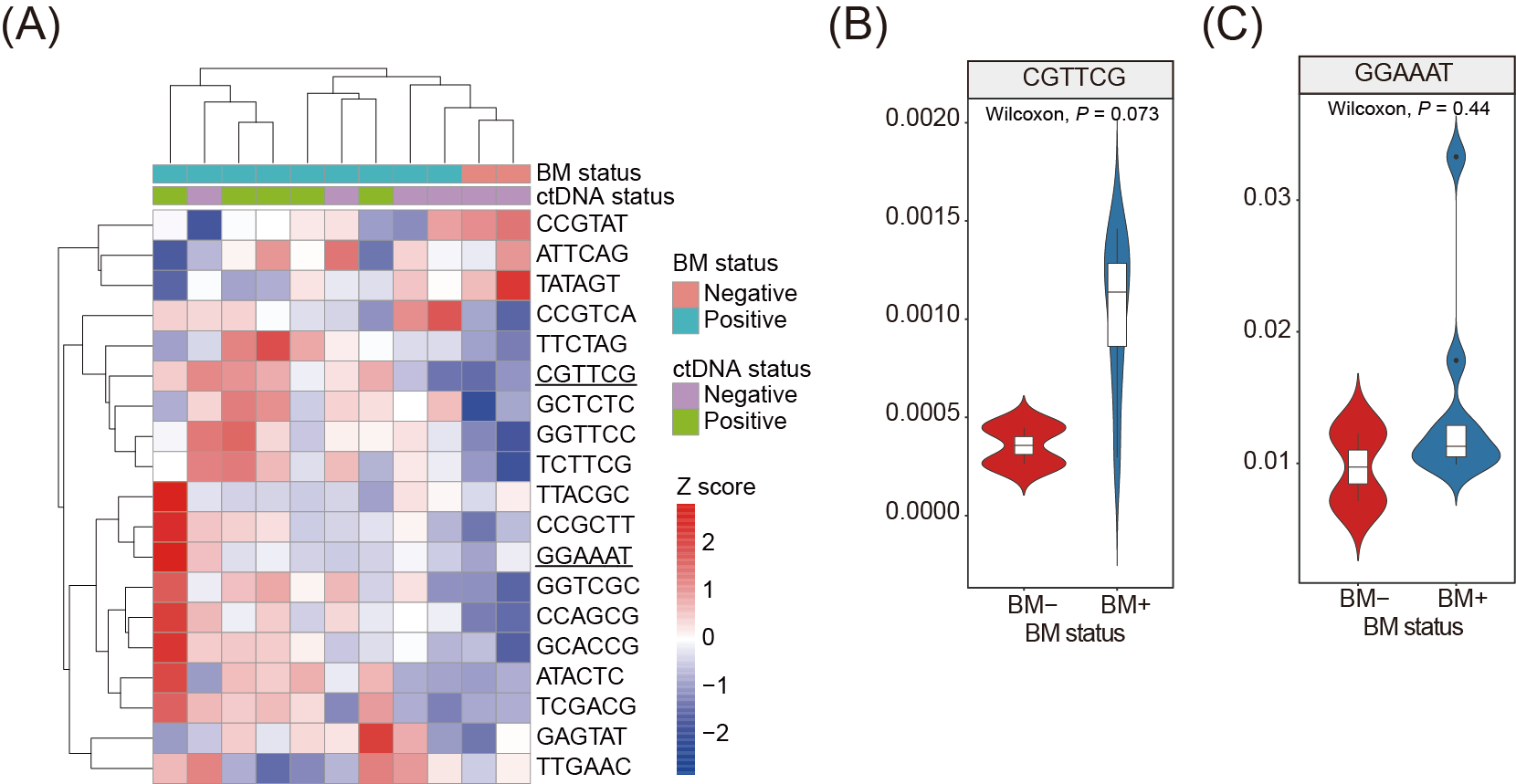


### Figure S5. Hierarchical clustering analysis of BPM in the testing cohort.

(A) Hierarchical clustering analysis of BPM with non-zero coefficients in the testing cohort (N=11). Underlined motifs (CGTTCG and GGAAAT) had the greatest positive and negative coefficients in the training cohort. (B and C) Violin plots showing frequencies between BM-negative (BM-) and BM-positive (BM+) patients in the testing cohort for the CGTTCG motif (B) and the GGAAAT motif (C) that contributed most significantly to the predictive model.

## Supplementary Tables

| **Table S1. Clinical characteristics of patients (N=81)** | | | | | |
| --- | --- | --- | --- | --- | --- |
| Characteristics | All  (N=81) | NEG (N=10) | NTP (N=9) | POS (N=62) | *P* value |
| Subgroup |  |  |  |  | ~ |
| NEG | 10 (12.3%) | ~ | ~ | ~ |  |
| NTP | 9 (11.1%) | ~ | ~ | ~ |  |
| POS | 62 (76.5%) | ~ | ~ | ~ |  |
| Sex |  |  |  |  | 0.408 |
| female | 43 (53.1%) | 5 (50.0%) | 3 (33.3%) | 35 (56.5%) |  |
| male | 38 (46.9%) | 5 (50.0%) | 6 (66.7%) | 27 (43.5%) |  |
| Diagnosis age |  |  |  |  | 0.03 |
| <60 | 41 (50.6%) | 3 (30.0%) | 8 (88.9%) | 30 (48.4%) |  |
| ≥60 | 40 (49.4%) | 7 (70.0%) | 1 (11.1%) | 32 (51.6%) |  |
| median (range) | 59 (38-82) | 59 (51-57) | 54 (38-66) | 60 (38-82) | |
| Sampling age |  |  |  |  | 0.017 |
| <60 | 38 (46.9%) | 3 (30.0%) | 8 (88.9%) | 27 (43.5%) |  |
| ≥60 | 43 (53.1%) | 7 (70.0%) | 1 (11.1%) | 35 (56.5%) |  |
| median (range) | 62 (38-84) | 65 (51-72) | 54 (38-66) | 63 (38-84) | |
| Histology |  |  |  |  | 0.041 |
| NSCLC | 66 (81.5%) | 6 (60.0%) | 6 (66.7%) | 54 (87.1%) |  |
| SCLC | 15 (18.5%) | 4 (40.0%) | 3 (33.3%) | 8 (12.9%) |  |
| Brain metastases type |  |  |  |  | <0.001 |
| parenchymal | 45 (55.6%) | 0 (0.00%) | 5 (55.6%) | 40 (64.5%) |  |
| parenchymal + others* | 17 (21.0%) | 0 (0.00%) | 1 (11.1%) | 16 (25.8%) |  |
| others* | 9 (11.1%) | 0 (0.00%) | 3 (33.3%) | 6 (9.68%) |  |
| negative | 10 (12.3%) | 10 (100%) | 0 (0.00%) | 0 (0.00%) |  |
| Brain metastases diagnosis method | |  |  |  |  |
| MRI | 78 (96.3%) | 10 (100%) | 9 (100%) | 59 (95.2%) | 1.000 |
| MRI + CT | 3 (3.7%) | 0 (0.00%) | 0 (0.00%) | 3 (4.8%) |  |
| CSF ctDNA status |  |  |  |  | 0.026 |
| negative | 28 (34.6%) | 7 (70.0%) | 4 (44.4%) | 17 (27.4%) |  |
| positive | 53 (65.4%) | 3 (30.0%) | 5 (55.6%) | 45 (72.6%) |  |
| Smoking history |  |  |  |  | 0.006 |
| non-smoker | 54 (66.7%) | 4 (40.0%) | 4 (44.4%) | 46 (74.2%) |  |
| smoker | 25 (30.9%) | 4 (40.0%) | 5 (55.6%) | 16 (25.8%) |  |
| unknown | 2 (2.47%) | 2 (20.0%) | 0 (0.00%) | 0 (0.00%) |  |
| Karnofsky performance status | |  |  |  | 0.038 |
| ≥90 | 32 (39.5%) | 4 (40.0%) | 5 (55.6%) | 23 (37.1%) |  |
| 50-80 | 47 (58.0%) | 4 (40.0%) | 4 (44.4%) | 39 (62.9%) |  |
| unknown | 2 (2.47%) | 2 (20.0%) | 0 (0.00%) | 0 (0.00%) |  |
| Sampling stage |  |  |  |  | <0.001 |
| IV | 74 (91.4%) | 5 (50.0%) | 7 (77.8%) | 62 (100%) |  |
| unknown | 7 (8.64%) | 5 (50.0%) | 2 (22.2%) | 0 (0.00%) |  |
| CSF cytology |  |  |  |  | 0.738 |
| negative | 64 (79.0%) | 8 (80.0%) | 8 (88.9%) | 48 (77.4%) |  |
| positive | 7 (8.64%) | 0 (0.00%) | 0 (0.00%) | 7 (11.3%) |  |
| unknown | 10 (12.3%) | 2 (20.0%) | 1 (11.1%) | 7 (11.3%) |  |
| Treatment before BM diagnosis | |  |  |  | 0.001 |
| yes | 53 (65.4%) | 8 (80.0%) | 9 (100%) | 36 (58.1%) |  |
| no | 25 (30.9%) | 0 (0.00%) | 0 (0.00%) | 25 (40.3%) |  |
| unknown | 3 (3.70%) | 2 (20.0%) | 0 (0.00%) | 1 (1.61%) |  |
| NEG, BM-negative patients; NTP, patients whose BM status turned from negative to positive during the follow-up; POS, BM-positive patients at CSF sampling. *P* values were calculated to compare data among three patient subgroups using Fisher’s exact tests. *Others include leptomeningeal metastasis, spinal cord pial metastasis, leptomeningeal metastasis+spinal cord pial metastasis, and intramedullary metastasis. | | | | | |

| **Table S2. Univariate analyses of features associated with patient survival** | | |
| --- | --- | --- |
| Factor | HR (95% CI) | *P* value* |
| Sex (female) | 1.3 (0.81-2.1) | 0.27 |
| Sampling age (>60) | 0.93 (0.58-1.5) | 0.76 |
| Smoking (former/current smokers) | 0.9 (0.5~1.5) | 0.73 |
| Karnofsky performance status (≥90) | 1.3 (0.8~2.2) | 0.25 |
| NRF2 | 1 (0.32-3.3) | 0.96 |
| TGFβ | 1.6 (0.65-4.1) | 0.29 |
| MYC | 1.5 (0.6-3.5) | 0.36 |
| NOTCH | 1 (0.5-2.1) | 0.97 |
| WNT | 0.6 (0.3-1.3) | 0.20 |
| ­PI3K | 0.7 (0.4-1.2) | 0.18 |
| Cell cycle | 1.3 (0.7-2.1) | 0.39 |
| TP53 | 1 (0.6-1.6) | 0.92 |
| RTK/RAS | 1.1 (0.7-1.9) | 0.60 |
| Hippo | 1.3 (0.3-5.2) | 0.75 |
| Damage Sensing | 1.2 (0.4-3.8) | 0.79 |
| Homologous Recombination (HR) | 1.5 (0.6-3.5) | 0.34 |
| Non-homologous End Joining (NHEJ) | 2.3 (0.7-7.4) | 0.16 |
| Nucleotide Excision Repair (NER) | 1.3 (0.4-4.2) | 0.65 |
| Mismatch Repair (MR) | 1.5 (0.5-4.1) | 0.45 |
| DDR core pathway | 1.5 (0.9-2.7) | 0.14 |
| Fanconi anemia (FA) | 2.4 (1-5.7) | **0.04** |
| *Bold represents significant *P* values based on the log-rank test. | | |
